# Learning curve analysis of transvaginal natural orifice transluminal endoscopic hysterectomy

**DOI:** 10.1186/s12893-019-0554-0

**Published:** 2019-07-10

**Authors:** Chin-Jung Wang, Justina Go, Hui-Yu Huang, Kai-Yun Wu, Yi-Ting Huang, Yu-Cheng Liu, Cindy Hsuan Weng

**Affiliations:** 1 Department of Obstetrics and Gynecology, Chang Gung Memorial Hospital at Linkou, Kweishan, Taoyuan, Taiwan; 2 Chang Gung University College of Medicine, Taoyuan, Taiwan; 3 Department of Obstetrics and Gynecology, Chinese General Hospital and Medical Center, Manila, Philippines; 4 Department of Obstetrics and Gynecology, Chang Gung Memorial Hospital at Taipei, Taipei, Taiwan

**Keywords:** Cumulative sum method, Hysterectomy, Learning curve, Natural orifice transluminal endoscopic surgery, Uterus

## Abstract

**Background:**

No data are available to assess the learning curve for transvaginal natural orifice transluminal endoscopic hysterectomy for non-prolapsed uteri in benign gynecologic diseases. The lack of exposure to transvaginal natural orifice transluminal endoscopic hysterectomy during training, in addition to a poorly defined learning curve, further deters interested physicians from applying this technique to daily practice. The aim of this study was to evaluate the learning curve and perioperative outcome of transvaginal natural orifice transluminal endoscopic hysterectomy by an experienced endoscopist.

**Methods:**

A total of 240 cases of transvaginal natural orifice transluminal endoscopic hysterectomies with or without adnexectomy for various benign gynecologic diseases were included. Demographic data and various perioperative parameters were reviewed from the prospectively collected database. Operative time was set as a surrogate marker for surgical competency. The learning curve was evaluated using the cumulative sum method.

**Results:**

The overall mean operative time (OT) was 76.5 min ± 22.4. Four unique phases of the learning curve were derived using cumulative sum analysis: the mean OT of phase I (the initial learning curve of 20 cases) was 86.3 ± 23.7 min, phase II (acquisition of competence of 80 cases) was 71.0 ± 21.4 min, phase III (proficiency and plateau of 80 cases) was 76.0 ± 20.4 min, and phase IV (post-learning in which more challenging cases were managed) was 81.3 ± 23.6 min. No major complications were encountered. One case in phase III converted to laparoscopy due to difficulty in performing anterior colpotomy.

**Conclusion:**

Our data demonstrated four distinct phases of the learning curve of transvaginal natural orifice transluminal endoscopic hysterectomy. In a well-trained endoscopist, surgical competence in transvaginal natural orifice transluminal endoscopic hysterectomy can be reached after 20 cases.

**Electronic supplementary material:**

The online version of this article (10.1186/s12893-019-0554-0) contains supplementary material, which is available to authorized users.

## Background

In recent years, the innovation of natural orifice transluminal endoscopic surgery (NOTES) has paved the way for a completely scarless surgery in the sense that no skin incisions are made. This technique utilizes the natural orifices of the body such as the mouth, anus, and vagina as the surgical channel for endoscopy and has aroused profound interest among surgeons in different fields of specialization worldwide. NOTES has the advantages of eliminating incision-related complications and less postoperative pain [1, 2].

At our institution, a tertiary referral and teaching medical center, gynecologic endoscopists have performed tubal sterilization, adnexal surgery, and hysterectomy using the techniques of transvaginal NOTES since 2010 [3, 4]. An earlier study done by this author (Wang, CJ) showed that transvaginal natural orifice transluminal endoscopic hysterectomy (tVNOTEH) can be performed safely for large and non-prolapsed uteri [5]. tVNOTEH has broadened the indications for vaginal hysterectomy (VH) and helped overcome its limitations. As applications for tVNOTEH is increasing, it is deemed important to assess the learning curve of this novel procedure. To our knowledge, no study has yet been published on the learning curve for tVNOTEH.

The present study attempted to evaluate the learning curve in tVNOTEH with or without bilateral salpingo-oophorectomy based on 240 consecutive cases handled by a single surgeon at a single institution.

## Methods

In this retrospective cohort study, we reviewed 240 patients (age range, 33–70 years; mean, 46.6 ± 4.9 years) who underwent tVNOTEH performed by a minimally invasive gynecologic surgeon (CJW) at Chang Gung Memorial Hospital at Linkou between April 2011 and February 2016. All patients underwent thorough clinical evaluation, including detailed medical histories, pelvic examinations and scheduled for tVNOTEH if uterine fibroids, adenomyosis, cervical intraepithelial neoplasm grade 3, or endometrial complex hyperplasia with atypia were indicated. Exclusion criteria including a documented history of abdominal–pelvic surgery with adhesion formation, uterine prolapse (International Continence Society classification stage III or IV), suspected severe endometriosis, and complete obliteration of the posterior Douglas pouch determined by pelvic examination. A history of cesarean section and nullipara were not considered as contraindications for tVNOTEH. Before the operation, the patients were informed of the risks and benefits of tVNOTEH, including the potential need to switch to laparoscopy or laparotomy during the operation and the risks of intraoperative bleeding, need for blood transfusion, and possible adhesion formation. All subjects gave informed consent prior to the start of the study. The participants underwent bowel preparation on the morning of the surgery. Blood transfusion with packed red blood cells (2 units) was required if preoperative hemoglobin level below than 8 mg/dL. Intravenous cephalosporin prophylaxis was administered just before surgery.

Preoperative clinical and demographic characteristics including age, body mass index (BMI), and parity were obtained. Similarly, OT, specimen (uterus) weight, estimated blood loss (EBL), postoperative hemoglobin, postoperative stay, requirement of blood transfusion, and any perioperative complications (fever, bowel injury, or genitourinary tract injury) were recorded. The study was approved by the Institutional Review Board of Chang Gung Memorial Hospital.

### tVNOTEH technique

The tVNOTEH operative technique has been described in detail elsewhere [3, 5]. In brief, the patient was placed in the dorsolithotomy Trendelenburg position. Bilateral lower extremities were wrapped in and protected by elastic bandages to prevent venous thromboembolism, and a Foley catheter was inserted to record fluid status and for urinary drainage. Under general anesthesia, traditional vaginal entry was performed. This involved creating the anterior and posterior colpotomies. The cardinouterosacral ligament complexes and parametrium were then dissected along the uterus to the level of the uterine artery. A single-port device consisting of a wound retractor (Alexis, Small; Applied Medical Resources Corp., Rancho Santa Margarita, CA) and a surgical glove where two 10-mm and one 5-mm sheaths were inserted through the slits created in the thumb, middle, and little fingertips was then placed transvaginally. Several instruments including a 0^°^ 10-mm laparoscope containing a video camera, conventional rigid straight laparoscopic grasper, a single-tooth tenaculum, and the LigaSure vessel sealer (Covidien, Mansfield, MA) were utilized throughout the surgery to facilitate various procedures such as holding, cutting, exploring, and dissecting (Additional file 1). The detached uterus was removed vaginally by detaching the glove from the wound retractor that was left in place. If the size of the specimen was larger than the diameter of the opening of the wound, a combination of vaginal bisection, coring and/or myomectomy was. After removal of the detached uterus, the glove was attached again, and the pneumoperitoneum was reestablished, and all pedicles were inspected to ensure hemostasis. Finally, the glove and wound retractor were removed, and the vaginal cuff was closed with 1–0 polyglycolic acid sutures (Vicryl; Ethicon Inc., Somerville, NJ, USA) (Additional file 2). Prior to the end of the surgery, cystoscopic evaluation was performed to examine for any unsuspected lower urinary tract injuries.


**Additional file 1:** The procedures of anterior-posterior colpotomy and single-port surgical device setting. (MP4 462337 kb)



**Additional file 2:** The procedures of hysterectomy and closure of vaginal cuff. (MP4 246685 kb)


### Statistical analysis

The learning curve of the tVNOTEH was measured as the OT over the time course of the study. Cumulative sum (CUSUM_OT_) analysis was used here as described by previous studies [6, 7]. The CUSUM calculated the total difference between the individual values and mean of all values. Using the OT of patients who were arranged in sequence, graphical information of the trend in the OT of consecutive procedures could be plotted. The CUSUM_OT_ for the 1st case was the difference between the OT for the 1st case and the mean OT for all patients. The CUSUM_OT_ of the 2nd case was the CUSUM_OT_ of the 1st value added to the difference between the OT of the 2nd case and the mean OT for all patients. The calculation was repeated until the last CUSUM_OT_ reached zero. Linear regression with log transformations was performed to determine the sign of the slope of regression.

Continuous variables were presented as descriptive statistics including mean and standard deviation. Discrete variables were presented as percentages. Continuous variables were compared using Kruskal-Wallis tests, while categorical values were compared using Pearson’s χ^2^ analysis or Fisher’s exact tests. All probability values were two sided. The significance level (alpha) was set at 0.05. All analyses were performed using PASW Statistics for Windows version 18.0 (SPSS, Inc., Chicago, IL, USA).

## Results

Table 1 summarizes the demographics, perioperative characteristics, postoperative outcomes, and pathological diagnoses of the 240 consecutive patients who underwent tVNOTEH. Most of the patients (143/240, 59.6%) had symptomatic uterine fibroids. All procedures were completed successfully with one laparoscopy conversion (case 155). The patient suffered from anterior wall fibroid, which interfered with the anterior colpotomy. Operative procedures included unilateral salpingectomy in 10 patients, bilateral salpingectomy in 10 patients, and adnexectomy in 7 patients. Mean OT was 76.5 ± 22.4 min (range, 35–150 min). Mean blood loss was 167.1 ± 144.5 mL (range, 20–900 mL). Thirteen of 240 (5.4%) patients received intraoperative blood transfusion.

**Table 1:** Overall patient characteristics (*n* = 240)

Characteristic	Value
Age (y)	46.6 ± 4.9 (33–70)
Body Mass Index (kg/m^2^)	24.2 ± 3.8 (16.4–40.6)
Parity	2.3 ± 0.9 (0–6)
Vaginal delivery	1.8 ± 1.2 (0–5)
No vaginal delivery^a^	53 (22.1)
Uterine weight (g)	409.9 ± 195.0 (35–1086)
Operating time (min)	76.5 ± 22.4 (35–150)
Blood loss (mL)	167.1 ± 144.5 (20–900)
Blood transfusion	13 (5.4)
Transient hematuria	31 (12.9)
Complication	5 (2.1)
Postoperative stay (days)	2.1 ± 0.4 (1–4)
Pathological diagnoses	
Adenomyosis	85 (35.4)
Uterine myoma	143 (59.6)
Cervical dysplasia	9 (3.8)
Endometrial hyperplasia	3 (1.3)

Data are presented as mean ± SD (range) or n (%)

^a^Include nulliparae and cesarean delivery

**Fig. 1:**
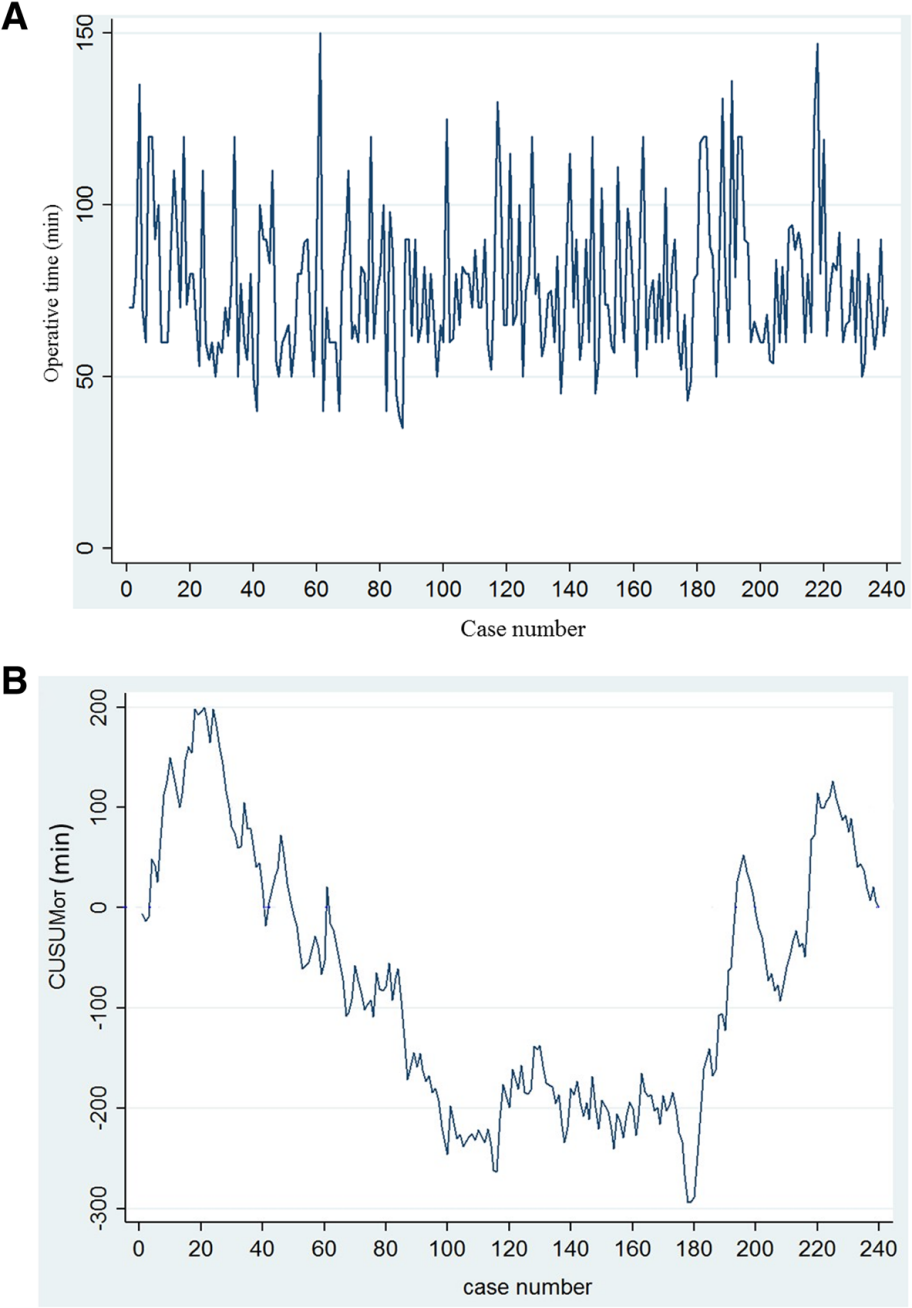
**a** Graph of raw operative time (OT) plotted against a chronological case number (240 consecutive patients). **b** Cumulative sum (CUSUM) of OT plotted against a case number

**Fig. 2:**
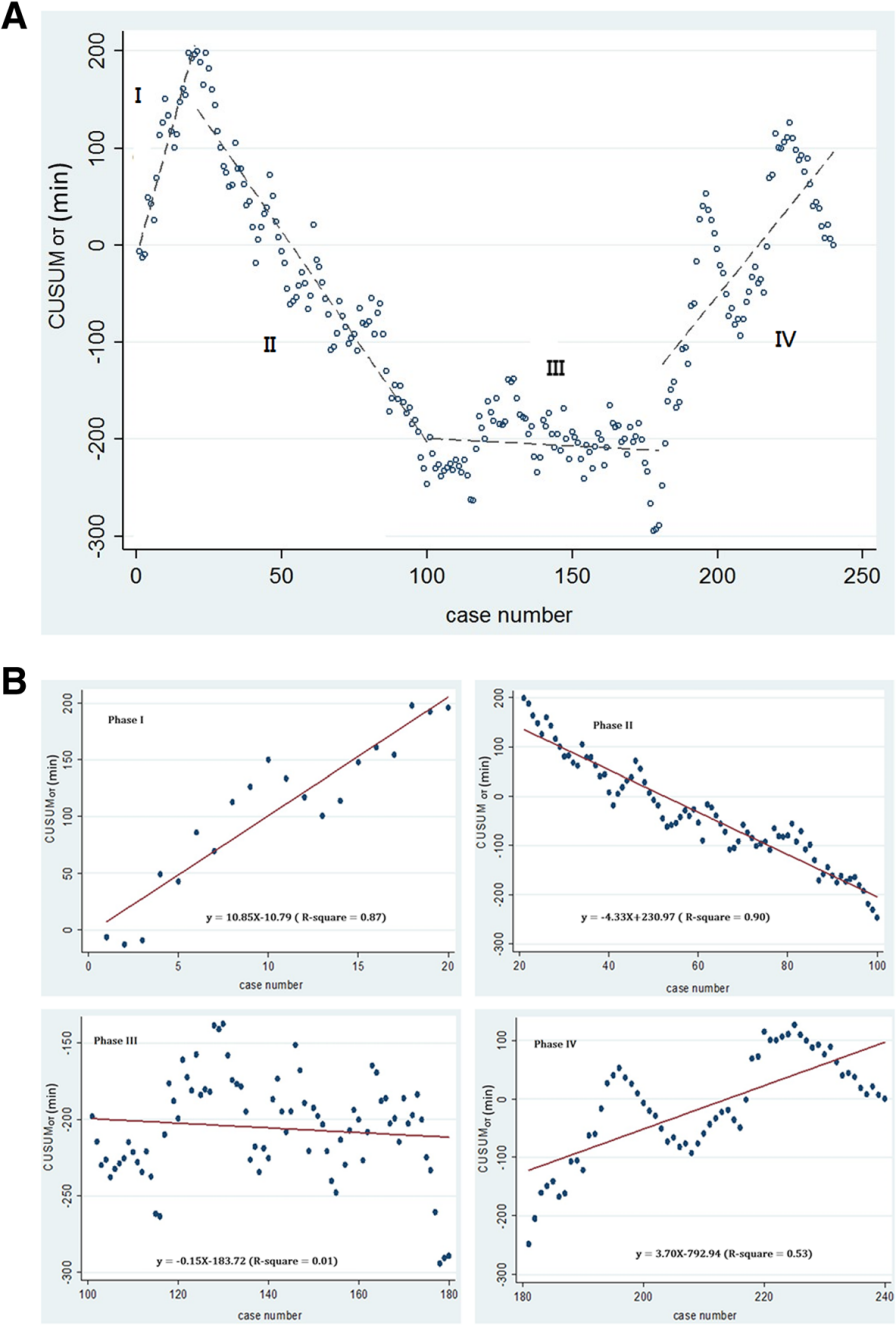
**a** The four phases of operative time in terms of the cumulative sum learning curve. **b** Lines of best fit for each phase. Phase I shows the initial learning phase. Phase II shows acquisition of competence phase after the initial 20 cases. Phase III shows the proficiency and plateau phase after 181 cases. Phase IV shows the post-learning phase

After getting the raw OT in each consecutive patient in chronological order (Fig. 1a), the learning curve by calculating the CUSUM_OT_ values was plotted in a graph as shown in Fig. 1b. We were able to divide four distinct phases in the graph: phase I, cases 1–20; phase II, cases 21–100; phase III, cases 101–180; and phase IV, cases 181–240 as shown in Fig. 2a. Figure 2b shows the lines of best fit for the four phases of the learning curve, designated here as the initial learning phase (phase I), acquisition of competence phase (phase II), proficiency and plateau phase (phase III), and post-learning phase (phase IV).

Comparative analyses of the patient characteristics, operative outcomes, and postoperative data showed no significant differences in terms of BMI, EBL, and postoperative stay among the four phases. Statistically significant differences were seen with regard to OT (*p* < 0.001), blood transfusion (*p* = 0.02), and complication (*p* = 0.039). OT of phases I and IV were significantly longer than that of phase II. There were significantly more cases with blood transfusion in phases I and III than in phase II (Table 2).

**Table 2:** Interphase comparisons of patient characteristics and peri/postoperative outcomes

	Phase I (*n* = 20, case 1–20)	Phase II (*n* = 80, case 21–100)	Phase III (*n* = 80, case 101–180)	Phase IV (*n* = 60, case 181–240)	*P* ^a^
Body Mass Index (kg/m^2^)	24.3 ± 3.6	24.0 ± 4.7	24.4 ± 4.1	23.9 ± 3.3	0.919
Uterine weight (g)	352.2 ± 159.9	381.3 ± 187.7	413.5 ± 204.0	459.8 ± 195.4	0.064
Operating time (min)	86.3 ± 23.7	71.0 ± 21.4	76.0 ± 20.7	81.3 ± 23.6	0.001
Blood loss (mL)	230.0 ± 165.8	175.9 ± 132.6	146.6 ± 138.7	161.5 ± 156.3	0.122
Blood transfusion	4 (1.7)	1 (0.4)	5 (2.1)	3 (1.3)	0.020
Complication	1 (0.4)	0 (0)	4 (1.7)	0 (0)	0.039
Postoperative stay (days)	2.2 ± 0.5	2.1 ± 0.4	2.0 ± 0.5	2.1 ± 0.3	0.669

Data are presented as mean ± SD or n (%)

^a^Kruskal-Wallis test

There were significant differences between the phase I-III (learning phase) and IV (post-learning phase) in terms of OT and uterine weight. Patients in the phase I-III were shorter OT (74.8 ± 21.5 min vs 81.3 ± 23.6 min, *p* = 0.041) and smaller uteri (395.2 ± 193.5 g vs 459.8 ± 195.4 g, *p* = 0.044) than patients in the phase IV. However, EBL and postoperative stay were similar between the phase I-III and IV.

There were significantly more complications in phase III than in phase I. In phase I, one patient had left infundibulopelvic ligament bleeding, which was controlled successfully via the vaginal route, but laparoscopy was performed for assurance. In phase III, four patients had complications: One patient had bladder injury which was repaired transvaginally; one patient had oozing from the stump of the right uterine artery, which was also controlled transvaginally; two patients developed a low-grade fever (< 38.5 °C) and had full recovery after fluid challenge and antibiotic therapy with cefamezine 1 g every 6 h and gentamicin 60 mg every 8 h for 1 to 3 days.

## Discussion

Our previous studies demonstrated the feasibility and applicability of tVNOTEH [3–5]. However, to improve and establish the standards of tVNOTEH, learning curves and quality of surgery must be evaluated and monitored persistently.

Results of this study showed that, in phase I, which is the initial learning phase, 20 cases were needed to acquire the basic skill in completing tVNOTEH with or without adnexectomy. In phase II, the phase of competence acquisition, about 80 cases were needed to consolidate the technique and acquire competence. In phase III, the proficiency and plateau phase, there was stabilization of OT in another 80 cases. In total, from phases I to III, there were 180 cases to fully master the technique and build up enough confidence to deal with more complex cases. In phase IV, the post-learning curve, the mean uterine weight was indeed heavier than phases I to III (459.8 ± 195.4 g vs 395.2 ± 193.5 g, *p* = 0.044) and this might reflect more challenging and difficult cases were handled after surgeon achieving proficiency. OT in the last 60 cases also demonstrated a positive shift accordingly (81.3 ± 23.6 min vs 74.8 ± 21.5 min, *p* = 0.041). In contrast to previous studies wherein an increase in OT was parallel to an increase in uterine weight [8], this study showed increasing CUSUM_OT_ only in phase IV wherein the mean uterine weight was 459.8 ± 195.4 g. Note that despite the increasing uterine weight, the CUSUM_OT_ was decreasing in phase II and plateaued in phase III. This implied that tVNOTEH might be more efficient for uterine weight of around 460 g or less.

The key to tVNOTEH is successful colpotomies. Complete obliteration of posterior cul-de-sac interfere with access to peritoneal cavity. In contrast, scarring of the anterior peritoneum (ex. caused by cesarean delivery) or in large and nonprolapsed uteri should not be excluded. Establishment of anterior colpotomy can be achieved by proper identification and dissection of the plane between bladder and cervix. Injection of diluted vasopressin into the vesicouterine fold [9] or access the vesicouterine peritoneum by using Sheth’s method [10] can facilitate accessing entering anterior Douglas pouch.

Since NOTES is a recent development in the field of minimally invasive surgery, no published studies are available to compare the learning curves of transvaginal NOTES-hysterectomy of different institutions. Yan et al. [11] reported on 16 patients who underwent hysterectomy by transvaginal NOTES in a retrospective cohort study. The mean duration of surgery (70.6 ± 12.8 min) was similar with ours (76.5 ± 22.4 min), but the hospital stay was longer (3.5 vs 2.1 days). One different aspect was that they amputated the uterine cervix (trachelectomy) after colpotomies were completed and then established single-port device placement, but we preserved the uterine cervix throughout entire surgery. Baekelandt [12] and Kale et al. [13] also reported their procedures in 10 and 7 patients, respectively. The greatest difference between this study compared with Yan et al. was that they used endoscopic techniques from the start to the end of the surgery, including colpotomies until uterus detachment. The mean OT in Baekelandt’s patients was 97 min and in Kale et al. was 73.1 min. Due to the limited case number and simply technical report, learning curves were not discussed by these studies.

A culmination of vaginal, endoscopic, and single-port access techniques were used during tVNOTEH surgery. The data regarding learning curves on traditional multiple-port and single-port laparoscopy-assisted vaginal hysterectomy (LAVH) are available in reported studies. A study from several surgeons in a single institution showed that to achieve competence in LAVH, 30 cases were needed [14]. Song et al. [15] reported that a well-trained surgeon in multi-port LAVH took 25 cases to attain proficiency in single-port LAVH and 75 cases to reach plateau. Although 20 cases were needed for competence in tVNOTEH and 100 cases were a doorsill for plateau as shown by this study, this did not mean that tVNOTEH could be easier to learn than LAVH and single-port LAVH. We should take the surgeon’s experience into consideration; the surgeon (CJW) who performed tVNOTEH here is an experienced laparoscopist.

The limitations to be considered in this study are as follows: First, the study was performed by an experienced surgeon proficient in both VH and LAVH. It may not be applicable to surgeons not familiar with either one or both procedures. Second, only one surgeon in a single institution was involved in the study. This might not represent the characteristic of the majority of the surgeons with different hospital setups, methods, and instruments at hand. Third, cases with additional adnexal surgery were included in this study. Therefore, the learning curve obtained here is not purely of hysterectomy alone.

## Conclusions

Our study has identified four phases in the learning curve of tVNOTEH. For an experienced surgeon adept in both laparoscopy and vaginal surgery, 20 cases comprised the initial learning curve, 100 for the acquisition of a competent phase, and 180 for the proficiency and acquisition of enough confidence to tackle more complex cases. Although OT starts to increase as uterine weight approaches 500 g, there was no associated increase in the complication rate. It is to be noted that the learning curve for transvaginal NOTES-hysterectomy may be shortened due to experienced laparoscopy. With rapid application of minimally invasive surgery in many hospitals, there might not be enough time for institutions to build up a step-by-step training program. Nevertheless, we believe these results may define the training assessment and impact the setting of NOTES and future trials.

## Data Availability

The datasets used and analyzed during the current study are available from the corresponding author on reasonable request.

## Abbreviations

BMI: Body mass index
CUSUM: Cumulative sum
EBL: Estimated blood loss
LAVH: Laparoscopy-assisted vaginal hysterectomy
NOTES: Natural orifice transluminal endoscopic surgery
OT: Operative time
tVNOTEH: Transvaginal natural orifice transluminal endoscopic hysterectomy
VH: Vaginal hysterectomy
